# Genome Sequences of *Gordonia terrae* Phages Benczkowski14 and Katyusha

**DOI:** 10.1128/genomeA.00578-16

**Published:** 2016-06-23

**Authors:** Welkin H. Pope, Matthew S. Benczkowski, Daryn E. Green, Melina Hwang, Bryan Kennedy, Bradley Kocak, Ellen Kruczek, Leon Lin, Matthew L. Moretti, Faith L. Onelangsy, Nadia Mezghani, Katherine A. Milliken, Chelsea L. Toner, Paige K. Thompson, Megan C. Ulbrich, Emily C. Furbee, Sarah R. Grubb, Marcie H. Warner, Matthew T. Montgomery, Rebecca A. Garlena, Daniel A. Russell, Deborah Jacobs-Sera, Graham F. Hatfull

**Affiliations:** Department of Biological Sciences, University of Pittsburgh, Pittsburgh, Pennsylvania, USA

## Abstract

Bacteriophages Katyusha and Benczkowski14 are newly isolated phages that infect *Gordonia terrae* 3612. Both have siphoviral morphologies with isometric heads and long tails (500 nm). The genomes are 75,380 bp long and closely related, and the tape measure genes (9 kbp) are among the largest to be identified.

## GENOME ANNOUNCEMENT

*Gordonia* spp. are common soil bacteria and are also associated with wastewater treatment plants (1). Several phages of *Gordonia* hosts have been isolated and sequenced, all of which have siphoviral morphologies, and many of which have unusually long tails (2–6). The Science Education Alliance-Phage Hunters Advancing Genomics and Evolutionary Science (SEA-PHAGES) program offers a course-based undergraduate research experience for advancing our understanding of bacteriophage diversity (7, 8).

Phages Benczkowski14 and Katyusha were isolated by enrichment of soil samples collected from Pittsburgh, PA, using *Gordonia terrae* 3612 as a host. Following plaque purification, amplification, and DNA extraction, the genomes were sequenced using Illumina MiSeq. Single-end 140-bp reads were assembled using Newbler, and both genomes assembled into a single major contig, with average coverages of 944-fold and 1,419-fold for Benczkowski14 and Katyusha, respectively. The genome coverage indicates that both have 1,172-bp direct terminal repeats and genome lengths of 75,380 bp. The genomes are almost identical and differ by just three single-nucleotide substitutions, but they are not closely related to previously reported phage genomes.

Protein-coding genes were predicted using Glimmer (9) and GeneMark (10), and functional assignments were made using BLASTP (11), HHpred (12), and Phamerator (13). Each genome has 99 putative protein-coding genes but no tRNA genes, and functional assignments could be made to fewer than 30% of the predicted genes. These include the virion structure and assembly genes, helicases, Holliday junction resolvases, HNH endonucleases, and WhiB regulators. Both genomes also encode a CobT-like protein.

Katyusha and Benczkowski14 have a strikingly large tape measure protein gene (9,084 bp), consistent with the length of the virion tails (approximately 500 nm). These are among the longest phage genes identified, and only slightly shorter than the tape measure protein gene of *Gordonia* phage GMA7 (9,141 bp [2]). Although GMA7 does not share extensive nucleotide sequence similarity with Katyusha and Benczkowski14, many of the virion structural proteins are related at the amino acid sequence level, including the portal, capsid maturation protease, major capsid subunit, major tail subunit, the tape measure protein, and some of the minor tail genes. The tape measure proteins share 50% amino acid identity with the tape measure protein of GMA7 and contain putative lytic transglycosylase domains, as reported for many mycobacteriophage tape measure proteins (14).

Benczkowski14, Katyusha, and GMA7 each have two genes located downstream of the virion structural gene operon coding for lysis functions, with one coding for a muramidase and a second coding for a peptidase. All three genomes also have two or more closely linked genes encoding putative protein products, at least one of which is anticipated to provide the holin function. We did not identify either integrase or putative repressor genes, which is consistent with a strictly lytic lifestyle.

### Nucleotide sequence accession numbers.

The genomes of Benczkowski14 and Katyusha are available from GenBank under the accession numbers KU963262 and KU963258, respectively.
